# Annual Meeting of Rock River Union Medical Society

**Published:** 1866-08

**Authors:** H. Utley, H. C. Donaldson

**Affiliations:** Pres’t.; Sec’y.


### ANNUAL MEETING OF ROCK RIVER UNION MEDICAL SOCIETY.

---

The physicians of Lee, Ogle, and Whiteside Counties, met at Morrison, Ill., on Wednesday, June 13th, it being the annual meeting of the above named Society, for mutual improvement. The meeting was called to order by the President, Dr. H. UTLEY.

The minutes of the last meeting were read and approved.

Dr. D. H. Law, of Dixon, and Dr. J. H. Page, of Como, were elected members; and, on motion, Dr. H. E. Dykeman was invited to participate in the proceedings of the Society, and to be considered a member, on presenting his credentials to the Board of Censors.

The Treasurer reported the current expenses of the Society all paid, and seventy-five cents in the treasury.

Dr. G. A. Bardwell reported a case of cerebro-spinal meningitis in a young lady. The Doctor reported the case, because of its presenting one peculiarity, *viz.*:—loss of vision in one eye, and a distinct milky appearance of the pupil of that eye. The disease having prevailed as an epidemic, during the past two years, in several localities in this region, and many of the members present having been called to treat cases of the same, therefore quite a lengthy discussion occurred, which was participated in by several of the members present.

On motion, the following resolution was unanimously adopted, *viz.*:—

*Resolved*, That, as Dr. D. C. Gould, of Sterling, Ill., who claims to be a "regular physician," and who made application to become a member of this Society, but failed to present his credentials or diploma; and has recently claimed to have discovered a specific for the cholera, which he advertises as worthy of approval by the medical profession, and which he is selling to the people; we take this occasion to state, that we, as a profession, and as individuals, do not endorse the course pursued by him; and we regard his so-called specific a humbug, and himself an imposter.

As officers for the ensuing year, the Society elected Dr. G. A. Bardwell, President; Dr. M. M. Royer, Vice-President; and Dr. H. C. Donaldson, Secretary and Treasurer.

The Society voted the forwarding of these minutes to both Chicago medical journals for publication, and also to the editors of our county papers, published at Dixon, Morrison, and Sterling.

Adjourned to meet at Sterling, on Wednesday, the 5th day of December, next.

H. UTLEY, M.D., *Pres't.*

H. C. Donaldson, M.D., *Sec'y.*

